# Whole-genome sequencing of a multidrug resistant *Escherichia coli* RSJ-SAU.Ec.2025 isolated from nasal swab of cat in Bangladesh

**DOI:** 10.1128/mra.00774-26

**Published:** 2026-09-03

**Authors:** Muhammad Abdul Mannan, Rubaya Sharmin Jhilik, S. M. Abdullah, Md. Abdullah Al Farhan, Mustafizur Rahman, Mohammad Jubair, Md. Morshedur Rahman, Md. Abu Ahsan Gilman, M. Nazmul Hoque, Md. Rezaul Karim

**Affiliations:** 1 Department of Microbiology & Parasitology, Faculty of Animal Science and Veterinary Medicine, Sher-e-Bangla Agricultural University (SAU) 54494, Dhaka, Bangladesh; 2 Genome Centre, Infectious Diseases Division, icddr,b, Dhaka, Bangladesh; 3 Molecular Biology and Bioinformatics Laboratory, Department of Gynecology, Obstetrics and Reproductive Health, Gazipur Agricultural University (GAU) 198780, Gazipur, Bangladesh; 4 AMR Reference Laboratory (Research), Bangladesh Livestock Research Institute (BLRI) 560480, Dhaka, Bangladesh; Fluxus Inc., Sunnyvale, California, USA

**Keywords:** *Escherichia coli*, whole-genome sequencing, cat, antimicrobial resistance

## Abstract

We report the whole-genome sequence of multidrug resistant *Escherichia coli* RSJ-SAU.Ec.2025 isolated from the nasal swab of a cat in Dhaka, Bangladesh. The assembled genome comprises 5,318,660 bp with 50.5% guanine-cystosine (GC) content, together with putative antibiotic-resistant genes.

## ANNOUNCEMENT

*Escherichia coli* is a gram-negative bacterium commonly found in the intestinal tract of humans and animals, as well as environmental sources, such as food, soil, and water (1, 2), posing a significant threat to public and veterinary health worldwide (3). Here, we report the whole-genome sequence of a multidrug-resistant *E. coli* strain RSJ-SAU.Ec.2025 recovered from the nasal swab of a calico cat in Dhaka, Bangladesh (23.8° N, 90.4° E).

The nasal swab sample was inoculated into 10 mL of nutrient broth (Liofilchem, Italy) and incubated overnight at 37°C. Following enrichment, one loopful inoculum of broth was then spread onto eosin-methylene blue (Liofilchem, Italy) agar and incubated at 37°C for 24 h. Presumptive *E. coli* colonies were subsequently subcultured on fresh EMB agar and MacConkey agar for purification. Initial identification of *E. coli* was performed based on colony morphology (green metallic sheen colored, circular shape on EMB and pinkish colored colonies at MacConkey) and Gram staining (gram-negative bacilli) (4).

Genomic DNA from the pure *E. coli* isolate RSJ-SAU.Ec.2025 was extracted from an overnight nutrient broth culture incubated at 37°C using the DNeasy Blood & Tissue Kit (Qiagen, Germany). DNA concentration was measured using the Qubit dsDNA quantification assay, yielding 13.7 ng/µL. Sequencing library was prepared from 300 ng of DNA using the Illumina DNA Prep Reagent Kit (Illumina, USA), and whole-genome sequencing (WGS) was performed on an Illumina MiSeq platform with a 2 × 250  bp paired-end protocol (5). Paired-end sequencing produced 1,016,276 reads. Raw sequencing reads were quality assessed using FastQC v0.11.3 (6). Trimmomatic v0.39.2 (7) was used to remove Illumina adapters from both forward (R1) and reverse (R2) reads. The genome was assembled using SPAdes v. 4.0.0 (8) into 362 contigs at least 500 nucleotides long, and the post-quality resulting assembly was checked with QUAST v5.3.0 (9). To assess assembly accuracy, genome completeness and contamination were determined with CheckM2 v1.1.0 (10), and average nucleotide identity (ANI) values were determined through FastANI v1.3 (11) to a reference *E. coli*. Genome annotation of *E. coli* RSJ-SAU.Ec.2025 was performed using the NCBI Prokaryotic Genome Annotation Pipeline (PGAP) v6.9 (12). Unless otherwise stated, default parameters were used for all software.

The genome size, guanine-cytosine (GC) content, genome coverage, and predicted gene numbers of *E. coli* RSJ-SAU.Ec.2025 are given in Table 1. Further analysis was further analysis of the assembled genome using AMRFinderPlus 3.11.20 (13), and ResFinder v4.7.2 (14) identified five putative AMR genes in the bacterial isolate, namely, *qnrB4*, *blaDHA-1*, *qacE*, *sul1*, and *dfrA7*. Mobile Element Finder v1.03 (15) identified three plasmids, namely, IncI1, ColRNAI, and IncFII(pHN7AB), and nine mobile genetic elements (MGEs). Functional subsystem analysis was performed using the RAST v2.0 server (16), and pathogenic potential was predicted with PathogenFinder v1.1 (17). Default parameters were applied for all analyses unless otherwise specified. Functional analysis revealed 388 subsystems, and a PathogenFinder score of 0.936 highlighted its potential relevance to both animal and human health. A summary of genome characteristics is provided in Table 1.

**TABLE 1:** Genomic features of *E. coli* RSJ-SAU.Ec.2025 isolated from nasal swab of a cat

Genomic features	*E. coli* RSJ-SAU.Ec.2025
GenBank accession	JBROPO000000000
BioSample accession	SAMN52085299
Assembled genome size (bp)	5,281,666
GC %	50.5
Coverage (×)	59
Genes (total)	5,453
CDSs (total)	5,397
Genes (RNA)	56
rRNAs	2, 2 (16S, 23S)
Partial rRNAs	2, 4 (16s, 23S)
tRNAs	45
ncRNAs	7
Pseudo genes (total)	205
CRISPR arrays	2
CheckM2 completeness	100
CheckM2 contamination	0.24
Average nucleotide identity (ANI)	98.7
N50	71,865
N90	9,496
auN	74,509
L50	25
L90	97

## Data Availability

The whole-genome sequence of the *Escherichia coli* strain has been deposited in GenBank under accession number JBROPO000000000. The sequence reads are deposited in the Sequence Read Archive (SRA) under accession number SRR35920869, and the BioProject accession number is PRJNA1336753.
